# CREB3L3 controls fatty acid oxidation and ketogenesis in synergy with PPARα

**DOI:** 10.1038/srep39182

**Published:** 2016-12-16

**Authors:** Yoshimi Nakagawa, Aoi Satoh, Hitomi Tezuka, Song-iee Han, Kenta Takei, Hitoshi Iwasaki, Shigeru Yatoh, Naoya Yahagi, Hiroaki Suzuki, Yasumasa Iwasaki, Hirohito Sone, Takashi Matsuzaka, Nobuhiro Yamada, Hitoshi Shimano

**Affiliations:** 1 Department of Internal Medicine (Endocrinology and Metabolism), Faculty of Medicine, University of Tsukuba, Tsukuba, Ibaraki 305-8575, Japan; 2 International Institute for Integrative Sleep Medicine (WPI-IIIS), University of Tsukuba, Tsukuba, Ibaraki 305-8575, Japan; 3 Health Care Center, Kochi University, Kochi Medical School, Kochi, Kochi 780-8520, Japan; 4 Department of Hematology, Endocrinology and Metabolism, Niigata University Faculty of Medicine, Niigata, Niigata 951-8510, Japan; 5 Life Science Center, Tsukuba Advanced Research Alliance (TARA), University of Tsukuba, Tsukuba 305-8577, Japan

## Abstract

CREB3L3 is involved in fatty acid oxidation and ketogenesis in a mutual manner with PPARα. To evaluate relative contribution, a combination of knockout and transgenic mice was investigated. On a ketogenic-diet (KD) that highlights capability of hepatic ketogenesis, *Creb3l3*^−/−^ mice exhibited reduction of expression of genes for fatty oxidation and ketogenesis comparable to *Ppara*^−/−^ mice. Most of the genes were further suppressed in double knockout mice indicating independent contribution of hepatic CREB3L3. During fasting, dependency of ketogenesis on CREB3L3 is lesser extents than *Ppara*^−/−^ mice suggesting importance of adipose PPARα for supply of FFA and hyperlipidemia in *Creb3l3*^−/−^ mice. In conclusion CREB3L3 plays a crucial role in hepatic adaptation to energy starvation via two pathways: direct related gene regulation and an auto-loop activation of PPARα. Furthermore, as KD-fed *Creb3l3*^−/−^ mice exhibited severe fatty liver, activating inflammation, CREB3L3 could be a therapeutic target for NAFLD.

The common characteristics of metabolic disorders, such as obesity, diabetes, cardiovascular diseases, and fatty liver, impair nutrient homeostasis, which is tightly regulated by balancing energy production (e.g. ketogenesis, gluconeogenesis, and lipid synthesis) with energy utilization (e.g. lipid oxidation). As fasting progresses, metabolic substrates stored in white adipose tissue (WAT) are released into the circulation as glycerol and free fatty acids (FFA) and transported into the liver. The liver then adapts by increasing β-oxidation, which converts fatty acids into acetyl coenzyme A (acetyl-coA), and by increasing ketogenesis, which converts the resulting acetyl-CoA into ketone bodies.

The first ketone body formed from acetyl-CoA is acetoacetate (Acac), which can generate acetone via non-enzymatic decarboxylation, as well as β-OH butyrate in a reaction catalysed by D-β-hydroxybutryate dehydrogenase (BDH1). The rate of conversion from acetyl-CoA to these ketone bodies is limited by hydroxymethylglutaryl CoA synthase 2 (HMGCS2), which converts acetoacetyl-CoA to HMG-CoA. The production of ketone bodies as an alternative energy source is crucial for maintaining energy homeostasis during fasting, as they are used as the main energy source for peripheral tissue, particularly the brain.

The fatty acid oxidation process consists of three pathways: peroxisomal β-oxidation, mitochondrial β-oxidation, and ω-oxidation in the endoplasmic reticulum (ER). Although the substrate spectra of mitochondrial and peroxisomal β-oxidation partly overlap, an important distinction is that the mitochondria catalyse the β-oxidation of the bulk of the short (<C8), medium (C8–C12), and long (C14–C20) chain fatty acids (LCFAs), whereas β-oxidation in the peroxisomes preferentially shortens very long chain fatty acids (>C20) to LCFAs, which can then be further oxidized in the mitochondria. FA transport across the mitochondrial membrane is triggered by carnitine palmitoyltransferase 1a, liver (CPT1a) and carnitine palmitoyltransferase 1b, muscle (CPT1b), which are localized in the mitochondrial membrane.

The activation of peroxisome proliferator-activated receptor α (PPARα) by fatty acids promotes hepatic fatty acid oxidation and ketogenesis. Several genes are directly regulated by PPARα in the liver, including those encoding acyl-CoA oxidase (*Acox1*)^1^, which is involved in the peroxisomal β-oxidation of fatty acids; *Cpt1a*^2^,^3^, which transports fatty acids across the outer mitochondrial membrane; and *HMGCS2*^2^,^3^,^4^. Consequently, mice that lack PPARα accumulate copious amounts of hepatic triglycerides (TG) and become hypoketonaemic and hypoglycaemic during fasting and starvation^5^,^6^,^7^.

cAMP responsive element-binding protein 3-like 3 (CREB3L3) is a basic, liver-specific leucine zipper transcription factor belonging to the CREB/ATF family^8^. Under ER stress, CREB3L3 traffics to the Golgi apparatus where site 1 and 2 proteases cleave its amino-terminal portion to induce the expression of genes that are responsible for the systemic inflammatory response^9^. *Creb3l3* expression is induced in fasted or insulin-resistant states, resulting in the accumulation of the nuclear form of CREB3L3^10^, which activates hepatic gluconeogenic genes, including phosphoenolpyruvate carboxykinase 1 (*Pck1*) and glucose-6-phosphatase (*G6pc*)^11^. *Creb3l3* deletion causes a defect in TG lipolysis in the blood, with *Creb3l3*^−/−^ mice exhibiting hypertriglyceridaemia as a result of inefficient catalysis of TG clearance by lipoprotein lipase (LPL); this is due to a reduction in the expression of the LPL coactivators Apolipoprotein c2 (*Apoc2*), *Apoa4*, and *Apoa5*, and upregulation of the LPL inhibitor *Apoc3*^12^,^13^. Thus, the defective expression of the enzymes that are required for lipolysis and lipid transport in the liver of *Creb3l3*^−/−^ mice may explain why mice that are fed either an atherogenic high-fat diet or normal chow exhibit hypertriglyceridaemia, reduced fat mass, body-weight gain, and massive steatosis^12^. When fed a methionine-choline-deficient diet or ketogenic diet, the deficiency of *Creb3l3* also develops hepatic steatosis^14^,^15^.

FGF21, a unique member of the FGF family with hormone-like actions^16^, is a key mediator of starvation that regulates lipolysis in WAT and increases fatty acid oxidation and ketogenesis in the liver^17^,^18^. There is some controversy over the effects of FGF21 on ketogenesis. Hotta *et al*.^19^ reported that FGF21 regulates lipolysis in WAT but is not required for ketogenesis and TG clearance in the liver, but Badman *et al*.^20^ showed that the adaptation to ketogenesis is impaired in *Fgf21*^−/−^ mice. FGF21 has been shown to have therapeutic effects on obesity-related metabolic disturbances, such as insulin resistance, diabetes, and hypertriglyceridaemia in *ob/ob* mice, diet-induced obese mice, and diabetic monkeys^21^,^22^. *Fgf21* expression is regulated by PPARα, which plays a key role in lipid oxidation, and is induced by fasting or ketogenic diet (KD) consumption^17^,^18^.

CREB3L3 and PPARα are activated in an auto-loop manner in response to starvation and, in turn, synergistically activate *Fgf21* expression^23^,^24^. Therefore, it is believed that these molecules have overlapping functions. In this study, we examined the role of CREB3L3 in energy metabolism during fasting by comparing *Creb3l3*^−/−^, *Ppara*^−/−^, and *Creb3l3*^−/−^*Ppara*^−/−^ mice.

## Results

### Effect of nutrient condition on the expression of *Creb3l3*

**Figure 1:**
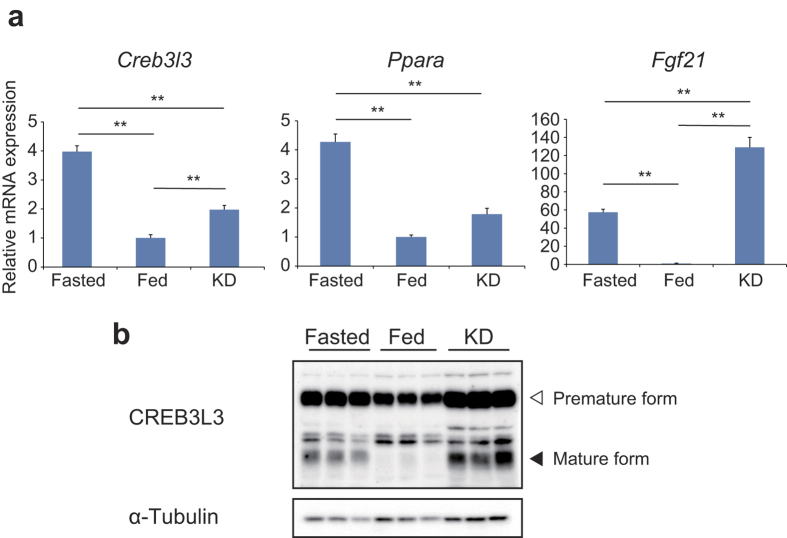
Effect of fasting and ketogenic diet on *Creb3l3* mRNA and the nuclear form of the CREB3L3 protein. Eight-week-old male wild type (WT) mice were fasted for 24 h, fed *ad libitum*, and fed a ketogenic diet (KD) for 3 days (*n* = 4–6 per group). (**a**) Gene expression in the liver of mice, as estimated using qPCR. Data are represented as mean ± SEM. Significant differences were determined by one-way ANOVA followed by Tukey-Kramer test and shown by *P < 0.05, **P < 0.01. (**b**) Immunoblotting of total liver lysates against the indicated antibodies. Full-length blots are presented in Supplementary Fig. 1.

First, we examined the expression levels of *Creb3l3* in the liver of wild type (WT) mice that had been fasted for 24 h (Fasted), fed *ad libitum* (Fed), or fed a KD for 3 days. Both the Fasted and KD groups had significantly higher expression levels of *Creb3l3* than did the Fed group (Fig. 1a), which led to higher protein levels of both mature and active forms of CREB3L3 in these groups (Fig. 1b, Supplementary Fig. 1). *Ppara* and *Fgf21* were also upregulated in the Fasted and KD groups (Fig. 1a). Thus, there was a positive correlation between the expression of *Creb3l3* and that of *Ppara* and *Fgf21* in response to the feeding conditions.

### KD-induced hepatic ketogenesis

**Figure 2:**
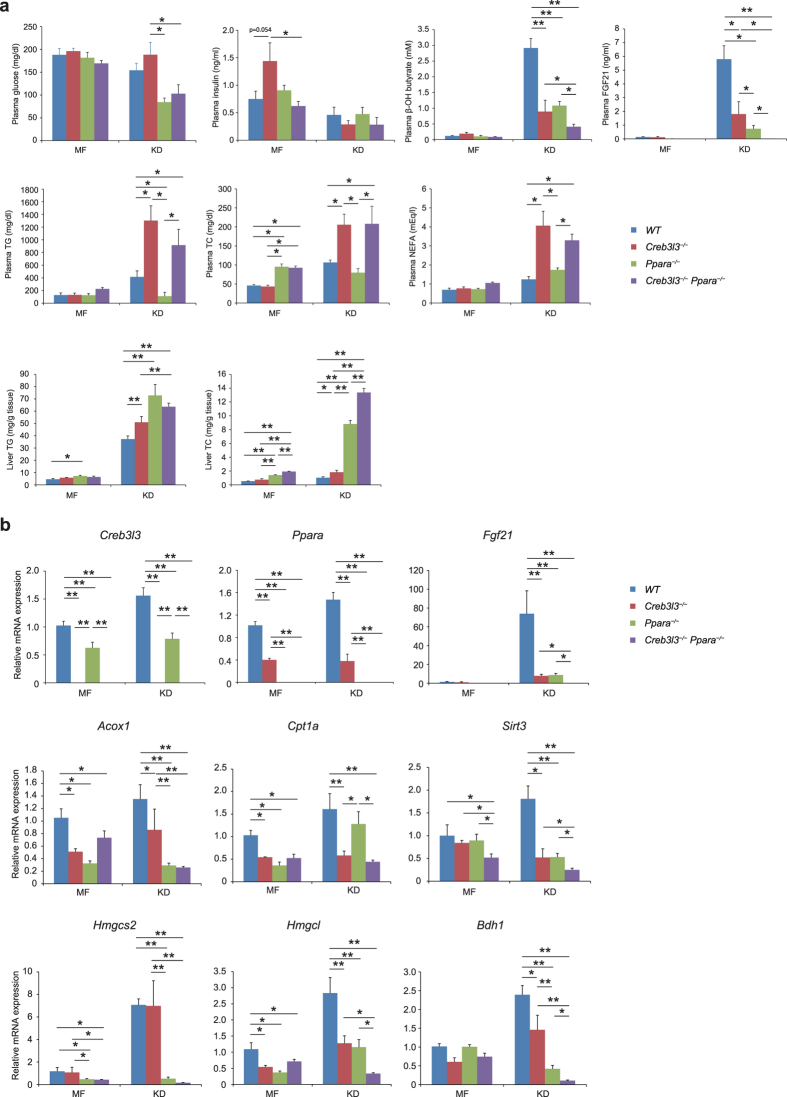
Metabolic parameters of wild type (WT), *Creb3l3*^−/−^, *Ppara*^−/−^, and *Creb3l3*^−/−^*Ppara*^−/−^ mice fed a ketogenic diet (KD) for 3 days. Eight-week-old male WT, *Creb3l3*^−/−^, *Ppara*^−/−^, and *Creb3l3*^−/−^*Ppara*^−/−^ mice were fed a medium-fat (MF) diet or KD for 3 days. (**a**) Plasma levels of glucose, insulin, β-OH butyrate, FGF21, triglycerides (TG), total cholesterol (TC), and free fatty acids (FFA). Liver TG and TC contents. (**b**) Gene expression in the liver of these mice, as determined using qPCR. All data are represented as mean ± SEM. Significant differences were determined by one-way ANOVA followed by Tukey-Kramer test and shown by *P < 0.05, **P < 0.01. *n* = 5–16 per group.

Mice that are fed on KD, a low-carbohydrate high-fat diet, have enhanced lipid oxidation and ketogenesis in the liver^25^. WT mice that had been fed KD for a short term (3 days) exhibited significantly increased levels of plasma ketone bodies. In both *Creb3l3*^−/−^ and *Ppara*^−/−^ mice, these KD-induced plasma ketone body levels were suppressed by 50% compared with WT mice (Fig. 2a). Plasma glucose levels were identical in all three types of mice when fed a moderate-fat (MF) diet. In contrast, when they were fed KD, *Ppara*^−/−^ mice showed significantly low levels compared with WT mice, while *Creb3l3*^−/−^ mice showed a trend of increase (Fig. 2a). Plasma TG, total cholesterol (TC), and FFA levels were markedly higher in *Creb3l3*^−/−^ KD-fed mice than in any other group, and thus increased in *Creb3l3*^−/−^*Ppara*^−/−^ mice (Fig. 2a). These findings indicate that the deficiency of *Creb3l3* mainly contributes to these changes. In contrast, liver TG and TC levels increased in *Creb3l3*^−/−^, *Ppara*^−/−^, and *Creb3l3*^−/−^*Ppara*^−/−^ mice (Fig. 2a). KD-fed induction emphasized the contribution of CREB3L3 along with PPARα and discriminated the effects of these factors on plasma glucose.

The expression of *Creb3l3* and *Ppara* was significantly downregulated in *Ppara*^−/−^ mice and in *Creb3l3*^−/−^ mice, respectively, that were fed both MF diet and KD (Fig. 2b). In response to carbohydrate depletion, expression of *Fgf21* significantly upregulated in KD-fed WT mice compared with normal chow-fed mice, but it completely blunted in *Creb3l3*^−/−^, *Ppara*^−/−^, and *Creb3l3*^−/−^*Ppara*^−/−^ mice (Fig. 2b). The expression of *Acox1* and *Hmgcs2*, which encode the rate-limiting enzymes for fatty acid oxidation and ketogenesis, were blunted in KD-fed *Ppara*^−/−^ and *Creb3l3*^−/−^*Ppara*^−/−^ mice. In addition, the expression of *Cpt1a* was significantly downregulated in *Creb3l3*^−/−^ KD-fed mice but unaffected in *Ppara*^−/−^ KD-fed mice. The expression of both *Hmgcl* and *Bdh1* was additionally downregulated in *Creb3l3*^−/−^*Ppara*^−/−^ mice compared with that in *Ppara*^−/−^ mice, indicating that CREB3L3 regulates these independent of PPARα. Thus, we speculated that *Cpt1a, Hmgcl*, and *Bdh1* are specifically candidate target genes of CREB3L3 in KD-fed mice. *Sirt3*, which is an activator for HMGCS2 to deacetylate it^26^, was downregulated in KD-fed *Creb3l3*^−/−^ and *Ppara*^−/−^ mice and further downregulated in *Creb3l3*^−/−^*Ppara*^−/−^ mice (Fig. 2b). These results indicate that CREB3L3 might regulate HMGCS2 enzymatic activity by Sirt3-mediated deacetylation.

### Metabolic parameters in WT, *Creb3l3* ^−/−^, *Ppara* ^−/−^, and *Creb3l3* ^−/−^ *Ppara* ^−/−^ mice

**Figure 3:**
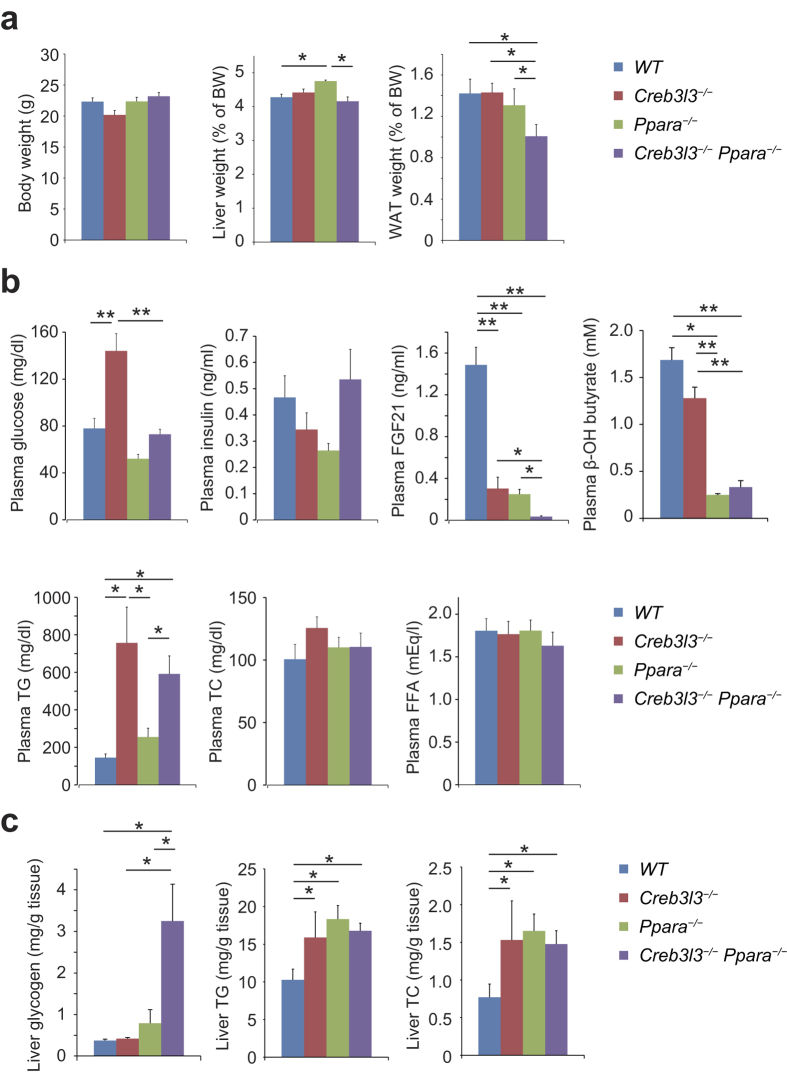
Metabolic parameters of wild type (WT), *Creb3l3*^−/−^, *Ppara*^−/−^, and *Creb3l3*^−/−^*Ppara*^−/−^ mice fasted for 24 h. Eight-week-old male WT, *Creb3l3*^−/−^, *Ppara*^−/−^, and *Creb3l3*^−/−^*Ppara*^−/−^ mice were fasted for 24 h. (**a**) Body, liver, and white adipose tissue (WAT) weights. (**b**) Plasma levels of glucose, insulin, FGF21, β-OH butyrate, triglycerides (TG), total cholesterol (TC), and free fatty acids (FFA). (**c**) Liver contents of glycogen, TG, and TC. Data are represented as mean ± SEM. Significant differences were determined by one-way ANOVA followed by Tukey-Kramer test and shown by *P < 0.05, **P < 0.01. *n* = 4–11 per group.

To determine the exact functions of CREB3L3 in comparison with PPARα, we evaluated the metabolic parameters of WT, *Creb3l3*^−/−^, *Ppara*^−/−^, and *Creb3l3*^−/−^*Ppara*^−/−^ mice in a fasted state. There was no difference in body weight between the four groups of mice. However, liver weight was the highest in *Ppara*^−/−^ mice, whereas WAT weight was significantly low in *Creb3l3*^−/−^*Ppara*^−/−^ mice (Fig. 3a). Plasma glucose levels were higher solely in *Creb3l3*^−/−^ mice, but there was no obvious difference in plasma insulin levels among the groups (Fig. 3b). Plasma β-OH butyrate levels were significantly lower in the three types of knockout (KO) mice than in WT mice, especially with further decrease in *Ppara*^−/−^ and *Creb3l3*^−/−^*Ppara*^−/−^ mice (Fig. 3b). Plasma FGF21 levels were also markedly lower in *Creb3l3*^−/−^ and *Ppara*^−/−^ mice than in WT mice and completely blunted in *Creb3l3*^−/−^*Ppara*^−/−^ mice (Fig. 3b), suggesting that *Fgf21* expression in a fasted state is entirely controlled by both CREB3L3 and PPARα.

*Creb3l3*^−/−^, but not *Ppara*^−/−^ mice, exhibited a marked increase in plasma TG levels in a fasted state, but there were no differences in the plasma TC and FFA levels between the groups of mice (Fig. 3b). There were also no differences in liver glycogen contents between WT, *Creb3l3*^−/−^, and *Ppara*^−/−^ mice; however, these were strongly higher in *Creb3l3*^−/−^*Ppara*^−/−^ mice (Fig. 3c). The KO mice had higher liver lipid contents (including TG and TC) than WT mice, but there was no difference between the KO mouse genotypes (Fig. 3c).

### Hepatic gene expression in WT, *Creb3l3* ^−/−^, *Ppara* ^−/−^, and *Creb3l3* ^−/−^ *Ppara* ^−/−^ mice

**Figure 4:**
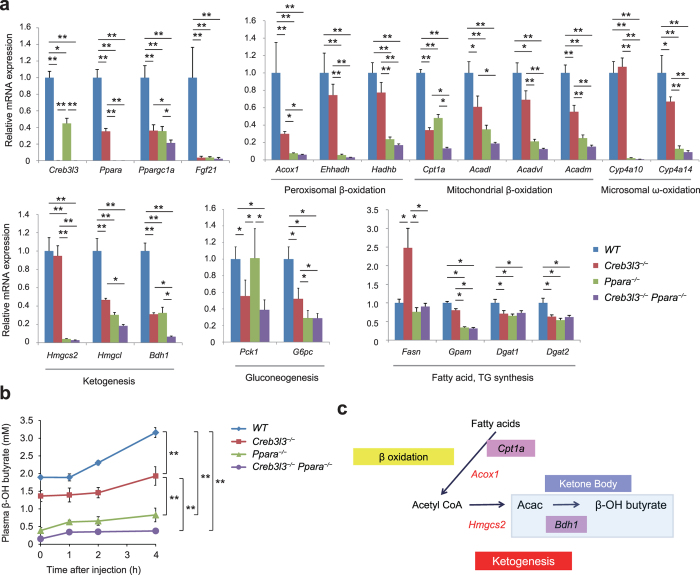
Gene expression in wild type (WT), *Creb3l3*^−/−^, *Ppara*^−/−^, and *Creb3l3*^−/−^*Ppara*^−/−^ mice fasted for 24 h. (**a**) Gene expression in the liver of 8-week-old male WT, *Creb3l3*^−/−^, *Ppara*^−/−^, and *Creb3l3*^−/−^*Ppara*^−/−^ mice fasted for 24 h, as determined using qPCR. Data are represented as mean ± SEM. Significant differences were determined by one-way ANOVA followed by Tukey-Kramer test and shown by *P < 0.05, **P < 0.01. *n* = 4–9 per group. (**b**) Ketogenic activity in 8-week-old male WT, *Creb3l3*^−/−^, *Ppara*^−/−^, and *Creb3l3*^−/−^*Ppara*^−/−^ mice fasted for 18 h and injected with sodium octanoate. Data are represented as mean ± SEM. Significant differences were determined by repeated-measures two-way ANOVA with Bonferroni post hoc t-test and shown by *P < 0.05, **P < 0.01. *n* = 4–7 per group. (**c**) A schematic diagram of how CREB3L3 affects *Cpt1a* in β-oxidation and *Bdh1* in ketogenesis, leading to the regulation of ketogenesis.

We determined the effect of CREB3L3 on the expression of genes involved in gluconeogenesis, β-oxidation, and ketogenesis in the liver by comparing gene expression in WT, *Creb3l3*^−/−^, *Ppara*^−/−^, and *Creb3l3*^−/−^*Ppara*^−/−^ mice in a fasted state (Fig. 4a). We found that *Creb3l3* mRNA decreased by approximately 50% in *Ppara*^−/−^ mice, and *Ppara* mRNA decreased by approximately 50% in *Creb3l3*^−/−^ mice presumably due to the mutual auto-loop system of the two factors^23^,^24^. Furthermore, the PPARα coactivator PPAR gamma coactivator 1α (*Ppargc1a*) decreased by 40% in both single KO and further in double KO mice, and *Fgf21* expression was completely blunted in all KO mice compared with WT mice, indicating that both factors are crucial for *Fgf21* expression.

*Cpt1a* and *Bdh1* demonstrated stronger and comparable suppression in *Creb3l3*^−/−^, respectively, with a further decrease in *Creb3l3*^−/−^*Ppara*^−/−^ mice (Fig. 4a), suggesting that *Cpt1a* and *Bdh1* are direct targets for CREB3L3 (Fig. 4b). Most of the genes related to peroxisomal β-oxidation, microsomal β-oxidation, and microsomal ω-oxidation were significantly downregulated in *Creb3l3*^−/−^ mice, but more markedly in *Ppara*^−/−^ mice with further suppression in both KO mice (Fig. 4a), suggesting that both factors are involved, but *Ppara* is more important in these gene expressions. *Cyp4a10* was not regulated by CREB3L3 but by PPARα. The gluconeogenic genes, including *Pck1* and *G6pc*, were downregulated in *Creb3l3*^−/−^ mice, which is consistent with previous reports^8^,^11^ as being a direct target, whereas only *G6pc* was downregulated in *Ppara*^−/−^ mice. Fatty acid synthase (*Fasn*) was significantly higher in *Creb3l3*^−/−^ mice.

In terms of ketogenic enzymes, the expression of *Hmgcs2* was blunted in *Ppara*^−/−^ and *Creb3l3*^−/−^*Ppara*^−/−^ mice and not in *Creb3l3*^−/−^mice, indicating that PPARα governs *Hmgcs2* expression. *Hmgcl* wass similarly decreased in all KO mice. There is the possibility that both factors co-operate its expression. The expression of *Bdh1* decreased in *Creb3l3*^−/−^ and *Ppara*^−/−^ mice and further in *Creb3l3*^−/−^*Ppara*^−/−^ mice, suggesting that this is a target for CREB3L3 as well as PPARα.

These findings indicated that CREB3L3 may play a role in regulating ketogenesis, although the contribution was less as compared to PPARα. Therefore, to confirm this, we directly estimated ketogenesis in mice by fasting the mice overnight and then intraperitoneally injecting them with sodium octanoate, a mitochondrial-permeable ketogenic substrate^27^. *Creb3l3*^−/−^ mice had significantly low β-OH butyrate levels compared with WT mice during this test (Fig. 4b), showing the impairment of hepatic ketogenesis activity in *Creb3l3*^−/−^ mice. *Ppara*^−/−^ and *Creb3l3*^−/−^*Ppara*^−/−^ mice showed a further decrease in β-OH butyrate levels during its test (Fig. 4b), indicating PPARα is more effective for ketogenesis.

### *In vivo* expression of *Bdh1* and *Cpt1a*

**Figure 5:**
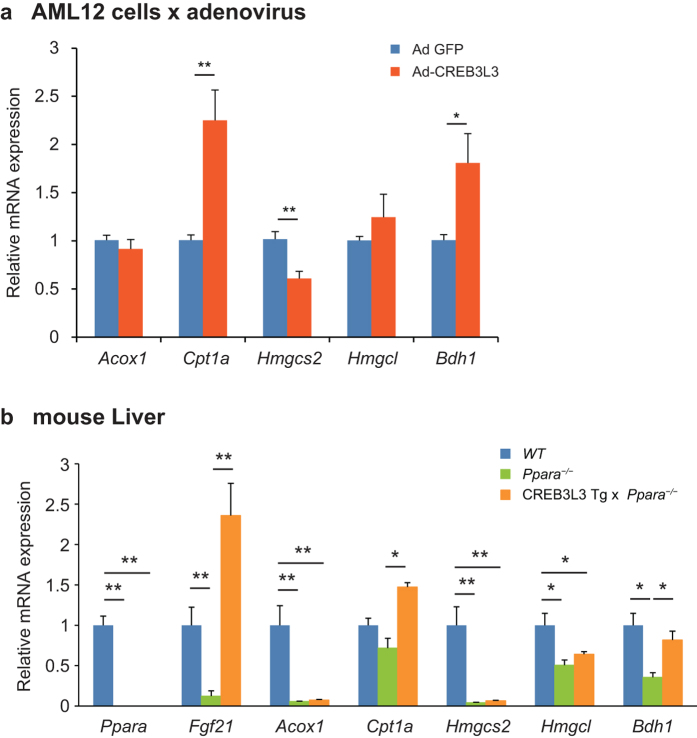
Effect of CREB3L3 on the expression of *Cpt1* and *Bdh1*. (**a**) Gene expression in AML12.2 mouse hepatoma cells 24 h after infection with an adenovirus encoding the active form of CREB3L3, as estimated using qPCR. Data are represented as mean ± SEM. Significant differences were determined by unpaired Student’s t test and shown by *P < 0.05, **P < 0.01. *n* = 3 per group. (**b**) Hepatic gene expression in 8-week-old male wild type (WT), *Ppara*^−/−^, and *Ppara*^−/−^ crossed with CREB3L3 Tg (*Ppara*^−/−^ × CREB3L3 Tg) mice in a fed state. Data are represented as mean ± SEM. Significant differences were determined by one-way ANOVA followed by Tukey-Kramer test and shown by *P < 0.05, **P < 0.01. *n* = 4 per group.

The *Creb3l3* knockout mouse studies outlined above identified *Cpt1a, Hmgcl*, and *Bdh1* as candidates of target genes for CREB3L3. To determine whether CREB3L3 directly regulates the expression of these genes, Ad-CREB3L3 was introduced into the mouse hepatoma cell line AML12.2 and quantitative polymerase chain reaction (qPCR) was performed. Ad-CREB3L3 significantly activated the expression of *Bdh1* and *Cpt1a* but did not affect the expression of any other β-oxidation and ketogenesis genes, such as *Acox1, Hmgcs2*, and *Hmgcl* (Fig. 5a).

To test our hypothesis that if *Cpt1a* and *Bdh1* are direct targets for CREB3L3, the reduction of them in *Ppara*^−/−^ mice might be dependent on CREB3L3. To determine the effects of CREB3L3 on the expression of both *Cpt1a* and *Bdh1*, we crossed *Ppara*^−/−^ mice with mice overexpressing the active form of human CREB3L3 in the liver (CREB3L3 Tg) to generate CREB3L3 Tg × *Ppara*^−/−^ mice, and then performed qPCR. The expression levels of the PPARα target genes *Acox1, Hmgcs2, Hmgcl*, and *Bdh1* were significantly downregulated in the liver of *Ppara*^−/−^ mice compared with WT mice (Fig. 5b). The expression of *Bdh1* and *Cpt1a* was significantly increased in CREB3L3 Tg × *Ppara*^−/−^ mice compared with that in *Ppara*^−/−^ mice (Fig. 5b). In contrast, there was no difference in the expression levels of the other genes, such as *Hmgcs2*, and *Acox1*, between *Ppara*^−/−^ mice and CREB3L3 Tg × *Ppara*^−/−^ mice (Fig. 5b). These results functionally confirmed that CREB3L3 could directly upregulate *Bdh1* and *Cpt1a* expression in the liver.

### Confirmation of *Cpt1* and *Bdh1* as CREB3L3-target genes using promoter analysis

**Figure 6:**
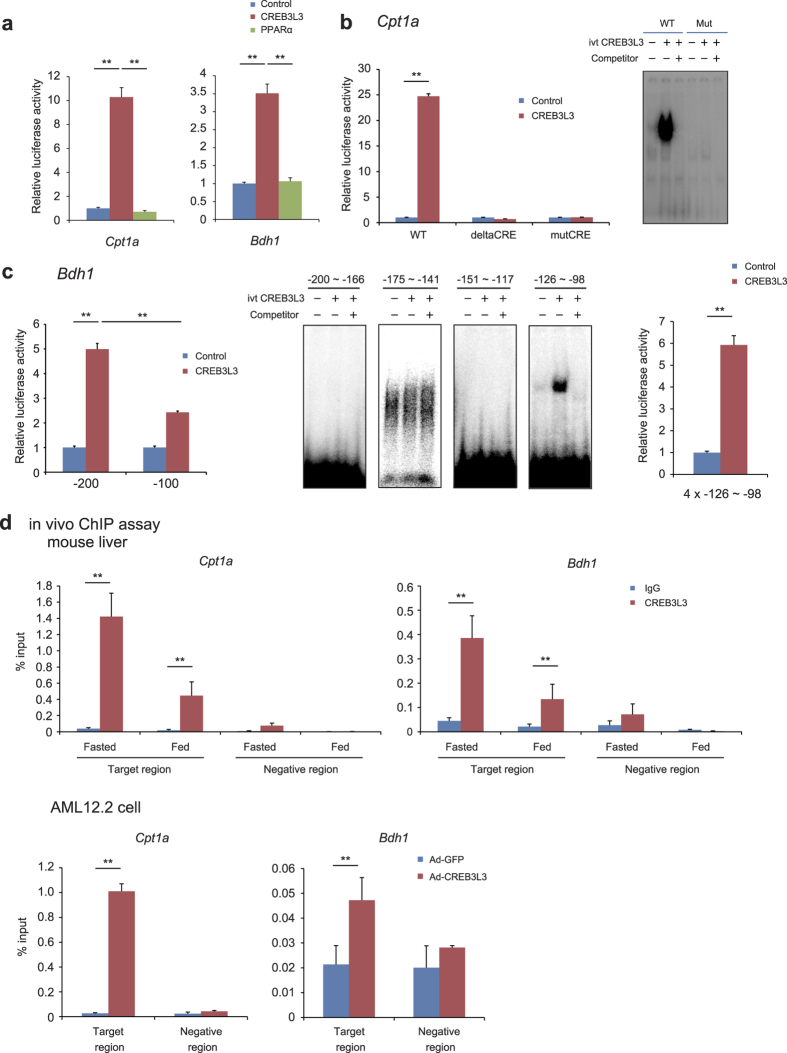
Promoter analysis of CREB3L3-induced *Cpt1a* and *Bdh1* expression. (**a**) Effects of CREB3L3 and PPARα on the *Cpt1a* and *Bdh1* promoter activity in mouse AML12.2 cells, as estimated using the luciferase promoter assay. Data are represented as mean ± SEM. Significant differences were determined by one-way ANOVA followed by Tukey-Kramer test and shown by *P < 0.05, **P < 0.01. *n* = 3 per group. (**b**) The effect of CREB3L3 on the *Cpt1a* promoter, as estimated using cAMP response element (CRE)-deleted and mutated vectors using the luciferase assay; the electrophoretic mobility shift assay (EMSA) showed that CREB3L3 directly bound to the CRE sequence. Data are represented as mean ± SEM. Significant differences were determined by unpaired Student’s t test and shown by **P < 0.01. *n* = 3 per group. (**c**) The effects of CREB3L3 on the *Bdh1* promoter, as estimated with a luciferase analysis using deletion constructs of the *Bdh1* promoter; the EMSA assay indicated that CREB3L3 directly bound to the region from −128 to −98 bp of the *Bdh1* promoter. The effect of CREB3L3 on the region from −128 to −98 bp of the *Bdh1* promoter, as estimated using the luciferase assay with 4-tandem region from −128 to −98 bp of the *Bdh1* promoter vector Data are represented as mean ± SEM. Significant differences were determined by one-way ANOVA followed by Tukey-Kramer test and shown by *P < 0.05, **P < 0.01. *n* = 3 per group. (**d**) Results of the chromatin immunoprecipitation (ChIP) assay demonstrating that CREB3L3 directly binds to the *Cpt1a* and *Bdh1* promoters *in vivo* in fasted and fed mice (*n* = 4 per group), and *in vitro* in AML12.2 cells infected with adenoviral GFP or the active form of CREB3L3 (*n* = 3 per group). Data are represented as mean ± SEM. Significant differences were determined by unpaired Student’s t test and shown by **P < 0.01.

To investigate whether CREB3L3 directly increases the expression of *Cpt1* and *Bdh1*, we performed a luciferase analysis using the *Cpt1a* and *Bdh1* promoters. CREB3L3 can bind to the cAMP response element (CRE) consensus sequence, which is a specific binding site of the CREB/ATF family of proteins^8^. We identified the CRE site at approximately −3 kb from the transcriptional initiation site of the mouse *Cpt1a* promoter. When the *Cpt1a* promoter luciferase vector containing this region was used in the luciferase assay, we found that it was CREB3L3 rather than PPARα that significantly activated the *Cpt1a* luciferase activity in the AML12.2 cells (Fig. 6a). We were unable to find the CRE sequence in the *Bdh1* promoter; however, we successfully cloned the −750-bp region of its promoter. As in the *Cpt1* promoter analysis, we found that CREB3L3, not PPARα, significantly activated *Bdh1* luciferase activity in the AML12.2 cells (Fig. 6a). These results indicate that CREB3L3 activates these promoters by regulating these regions independent of PPARα.

The CRE-deleted and mutated *Cpt1a* promoter luciferase vectors were completely blunted to activation by CREB3L3 (Fig. 6b). The electrophoretic mobility shift assay (EMSA) showed that CREB3L3 could bind to the CRE sequence in the *Cpt1a* promoter (Fig. 6b). We also constructed a series of deletion mutants of the *Bdh1* luciferase vectors, which showed that CREBL3 activated −200 bp of the *Bdh1* luciferase vector, but the −100-bp region was blunted to activation by CREB3L3. The EMSA assay showed that CREB3L3 bound to the region between −126 bp and −98 bp in the *Bdh1* promoter, but no other regions (Fig. 6c). CREB3L3 activated the luciferase vector containing 4x the region between −126 bp and −98 bp in the *Bdh1* promoter (Fig. 6c). The chromatin immunoprecipitation (ChIP) assay using the liver of mice showed that CREB3L3 directly bound to the *Bdh1* and *Cpt1a* promoter region more efficiently in mice that were in a fasted state than those that were in a fed state (Fig. 6d), which is consistent with the finding that the active form of the CREB3L3 protein is upregulated in fasted mice. Moreover, the ChIP assay using AML12.2 cells infected with adenoviral CREB3L3 showed that CREB3L3 directly bound to the *Bdh1* and *Cpt1a* promoter region (Fig. 6d). Thus, we confirmed *Bdh1* and *Cpt1a* as new target genes of CREB3L3.

### Effect of *Creb3l3* deficiency on KD-induced hepatosteatosis

**Figure 7:**
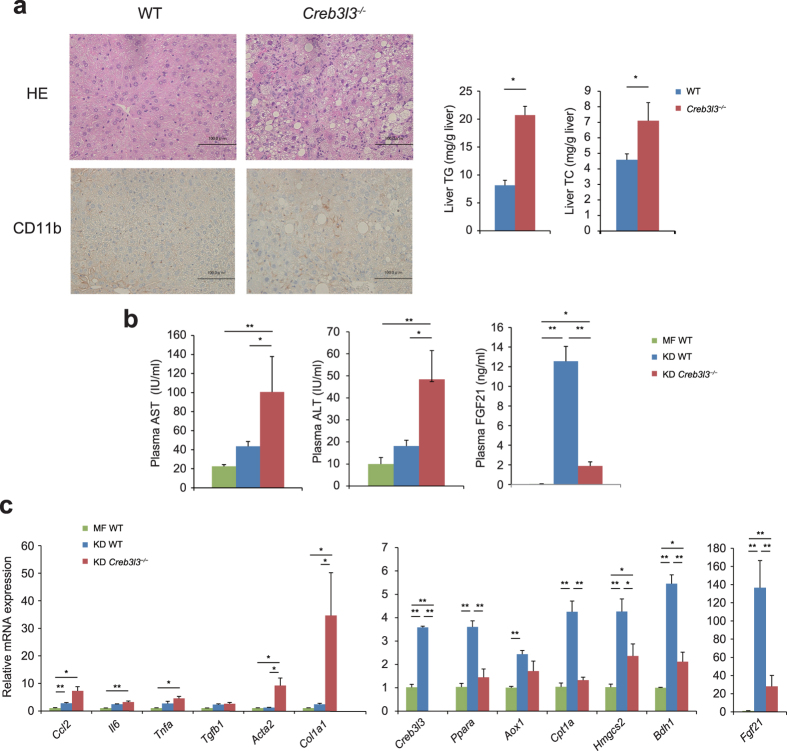
Effect of *Creb3l3* deficiency in the liver on ketogenic diet (KD)-induced fatty liver. Eight-week-old male wild type (WT) and *Creb3l3*^−/−^ mice were fed a medium-fat (MF) diet or KD for 4 weeks. (**a**) Histological analysis of liver sections from WT and *Creb3l3*^−/−^ mice that had been fed KD for 4 weeks, using haematoxylin and eosin staining and immunohistochemistry using the anti-CD11b antibody. Liver contents of triglycerides (TG) and total cholesterol (TC). Data are represented as mean ± SEM. Significant differences were determined by unpaired Student’s t test and shown by *P < 0.05. *n* = 5 per group. (**b**) Plasma aspartate aminotransferase (AST), alanine aminotransferase (ALT), and FGF21 levels, and (**c**) hepatic gene expression profiles of WT and *Creb3l3*^−/−^ mice that had been fed KD for 4 weeks. Data are represented as mean ± SEM. Significant differences were determined by one-way ANOVA followed by Tukey-Kramer test (**b,c**) and shown by *P < 0.05, **P < 0.01. *n* = 5–10 per group.

It has previously been shown that KD-fed mice exhibit suppressed lipogenic gene expression and increased expression of genes involved in fatty acid oxidation, ketogenesis, and inflammation of the liver^28^,^29^. Furthermore, long-term KD causes an injury pattern in WT mice similar to nonalcoholic fatty liver disease (NAFLD) phenotypes^28^,^30^. To clarify the effects of CREB3L3 on ketogenesis-related hepatosteatosis, WT and *Creb3l3*^−/−^ mice were fed MF or KD for 4 weeks, when WT mice does not show steatohepatitis. Histological analysis showed severe lipid accumulation and inflammation in the liver of *Creb3l3*^−/−^ mice compared with WT mice (Fig. 7a). Consistently, liver TG and TC levels were also significantly higher in *Creb3l3*^−/−^ mice than in WT mice (Fig. 7a). Immunohistochemistry using the anti-CD11b antibody as the macrophage marker showed that the liver sections from *Creb3l3*^−/−^ mice exhibited increased infiltration by inflammatory cells compared with WT mice (Fig. 7a), which resulted in increased levels of both plasma aspartate aminotransferase (AST) and alanine aminotransferase (ALT), which are markers of liver injury (Fig. 7b).

The hepatic expression of inflammatory marker genes, such as chemokine (C–C motif) ligand 2 (*Ccl2*), interleukin 6 (*Il6*), and tumour necrosis factor (*Tnf*), and fibrosis marker genes, such as alpha 2, smooth muscle, aorta (*Acta2*), and collagen, type I, alpha 1 (*Col1a1*), was significantly upregulated in KD-fed *Creb3l3*^−/−^ mice compared with that in MF diet-fed WT mice and KD-fed WT mice (Fig. 7c). These results confirmed that inflammation was enhanced in *Creb3l3*^−/−^ KD-fed mice. The expression of fatty acid oxidation genes *Ppara* and *Cpt1a*, and the ketogenesis genes *Hmgcs2, Bdh1, Fgf21*, and *Creb3l3* were also significantly upregulated in KD-fed WT mice compared with that in MF-fed WT mice (Fig. 7c) but significantly downregulated in KD-fed *Creb3l3*^−/−^ mice compared with that in KD-fed WT mice. Thus, the *Creb3l*3 deficiency suppressed fatty acid oxidation- and ketogenesis-related gene expression in the liver, even in mice that had been fed KD for a long time. Collectively, these data indicate that the dysfunction of fatty acid oxidation and ketogenesis in the liver of *Creb3l3*^−/−^ mice exacerbated the development of KD-induced hepatosteatosis.

## Discussion

CREB3L3 and PPARα have previously been shown to reciprocally activate each other and have overlapping functions such as changes of gene expression in the liver during fasting^23^,^24^. However, the functional differences between these proteins remained unclear. In this study, we investigated the direct effects of CREB3L3 on ketogenesis under various feeding conditions by comparing the phenotypes of WT, *Creb3l3*^−/−^, *Ppara*^−/−^, and C*reb3l3*^−/−^*Ppara*^−/−^ mice. We found that the deletion of either *Creb3l3* or *Ppara* resulted in considerable defects in the adaptation to energy starvation in mice. It should be noted that in either of the single KO mice, expression of the other gene was also decreased and thus additional effects of double deletion were further severe. Roughly, the deletion of *Ppara* largely blunted the hepatic gene expression of fatty acid oxidation and ketogenesis. It is conceivable that the synergistic interaction between CREB3L3 and PPARα should contribute to defected ketogenesis in *Creb3l3*^−/−^ mice as well as *Ppara*^−/−^ mice. We also found that CREB3L3 directly transactivates *Cpt1a* and *Bdh1*, thereby regulating ketogenesis independent of PPARα. C*reb3l3*^−/−^ mice, especially on a KD, accelerated KD-induced hepatosteatosis.

PPARα is ubiquitously expressed in not only the liver but some peripheral tissues. In fasting, PPARα induces lipolysis in WAT by activating ATGL and HSL^31^,^32^. In turn, fatty acids derived from lipolysis are important activators of PPARα^33^,^34^ and the substrates of fatty acid oxidation and ketogenesis in liver. Hepatic PPARα governs fatty acid oxidation and ketogenesis gene expression. Therefore, it is thought that the deficiency of adipose *Ppara* aggravates the defects of hepatic fatty acid oxidation and ketogenesis due to insufficiency of energy source supply from peripheral tissues and metabolic activity in the liver. On the other hand, as CREB3L3 is expressed in only the liver and small intestine, lipolysis in WAT in *Creb3l3*^−/−^ mice were comparative with that in WT mice. This could explain that reduced ketosis in *Creb3l3*^−/−^ mice was limited in fasting.

KD is a high-fat, low-carbohydrate diet containing approximately 80% fat. Although both fasting and a KD feeding induce ketogenesis, a KD feeding supplies energy, the sources of which are mainly from dietary fat, and not lipolysis in adipose tissue. Therefore, a KD feeding study can reflect on the functions in hepatic fatty acid oxidation and ketogenesis. The changes of fatty acid oxidation and ketogenesis in *Creb3l3*^−/−^ mice were comparable with those in *Ppara*^−/−^ mice, indicating both hepatic factors are equally important in this metabolism.

In this study, we identified that CREB3L3 directly regulates *Cpt1a*, the rate limiting molecule for FA transport into the mitochondria, and *Bdh1*, a component gene in the ketogenesis pathway. Moreover, CREB3L3 upregulates PPARα target genes via the regulation of both the gene expression and transcriptional activity of PPARα. The expression of *Cpt1a* was solely regulated by CREB3L3 in a KD feeding (Fig. 2), whereas it was regulated by both CREB3L3 and PPARα during fasting (Fig. 4a). These finding indicates that the PPARα-CREB3L3 interaction in a KD feeding is more important for the PPARα-mediated *Cpt1a* expression than in fasting.

CPT1a is one of the rate-limiting enzymes involved in mitochondrial β-oxidation, catalysing the esterification of long-chain acyl-CoAs to L-carnitine for transport into the mitochondria for fatty acid oxidation. Therefore, the mitochondrial carnitine system plays a crucial role in β-oxidation. The reduction in *Cpt1a* expression in the liver of *Creb3l3*^−/−^ mice inhibit the transport of fatty acids to the mitochondria and suppress fatty acid oxidation. KD contains approximately 80% fat, which mainly consists of long-chain acyl-CoAs. Thus, *Creb3l3*^−/−^ mice cannot catalyse the excess long-chain acyl-CoAs.

Ketogenesis is catalysed by the enzymes HMGCS2, HMGCL, and BDH1, which converts acetyl-CoA into β-OH butyrate. BDH1 is involved in the final step of this process, catalysing Acac to β-OH butyrate. In this study, we demonstrated that CREB3L3 directly regulates *Bdh1* expression. To increase the enzymatic activity of HMGCS2, deacetylation by SIRT3 is required^26^. The deficiency of *Creb3l3* reduced the expression of *Sirt3* in feeding a KD diet, indicating the reduction of *Hmgcs2* enzymatic activity in *Creb3l3*^−/−^ mice. Taken together, these results demonstrate that CREB3L3 plays a pivotal role in the ketogenic process that converts acetyl-CoA into β-OH butyrate in the liver.

These findings could propose that CREB3L3 contributes to ketogenesis; however, in the ketogenesis activity test, the deficiency of *Creb3l3* did not show the predicted loss of its activity. Because its test uses sodium octanoate as a substrate, octanoate can cross the mitochondrial membranes independently of CPT1a^35^. Therefore, the differences of *Cpt1a* expression between *Creb3l3*^−/−^ mice and *Ppara*^−/−^ mice could not reflected a change in the ketogenesis activity (Fig. 4b). The deficiency of *Ppara* had the further decrease of other fatty acid oxidation and ketogenesis gene expression compared with the deficiency of *Creb3l3*, thereby further decreasing the ketogenesis activity.

It has previously been shown that *Fgf21*^−/−^ mice exhibit impaired adaptation to ketosis^20^. In the present study, we found that the expression of hepatic *Fgf21* was blunted in both *Creb3l3*^−/−^ and *Ppara*^−/−^ mice, which could at least partially explain why the *Creb3l3* deficiency led to impaired ketogenesis.

It is well established that hepatic fatty acid oxidation disorders, including a deficiency in PPARα, lead to NAFLD^5^,^6^,^7^,^36^. High fat feeding of mice with insufficiency of ketogenesis results in NAFLD^37^. Consistently, the deficiency of *Creb3l3* developed severe hepatic steatosis.

In summary, our findings indicate that CREB3L3 regulates ketogenesis via two pathways: (1) by directly activating *Ppara* expression to activate PPARα-induced genes related to fatty acid oxidation and ketogenesis and (2) by directly activating *Cpt1a* and *Bdh1* expression. We propose that CREB3L3 co-operates with PPARα by directly and indirectly regulating the expression of genes involved in fatty acid oxidation and ketogenesis. In particular, CREB3L3 plays a role in exogenous (dietary) fatty acid homeostasis, while PPARα plays dual roles in exogenous and endogenous (released from lipolysis in adipose tissues) fatty acid homeostasis. Individuals with NASH have been shown to have lower levels of ketone bodies than individuals with simple steatosis^38^, and studies on humans have revealed that the levels of plasma ketone bodies is negatively correlated with the pathology of NASH, suggesting a reduction in ketone body metabolism in individuals with NASH^38^. Thus, as ketogenesis prevents diet-induced fatty liver injury and hyperglycaemia^37^, CREB3L3 may represent a new therapeutic target for NAFLD.

## Experimental Procedures

### Animals

Eight-week-old male C57/BL6J (WT) mice were obtained from CLEA Japan, and B6;129S4-*Ppara*^*tm1G*^*°*^*nz*^/J (*Ppara*^−/−^)^39^ and *Creb3l3*^*tm1.1Sad*^/J (*Creb3l3*^−/−^)^40^ mice were purchased from Jackson Laboratory. To generate the active form of human CREB3L3 transgenic (CREB3L3 Tg) mice, cDNA encoding the rat *Pck1* promoter^41^, human CREB3L3 (1–320 aa), and the 3′ polyadenylation signal of human growth hormone were microinjected into C57BL6J eggs^24^. To generate *Creb3l3*^−/−^*Ppara*^−/−^ mice, *Creb3l3*^−/−^ mice were crossed with *Ppara*^−/−^ mice; and to generate *Ppara*^−/−^ × CREB3L3 Tg (*Ppara*^−/−^CREB3L3 Tg) mice, *Ppara*^−/−^ mice were crossed with CREB3L3 Tg mice.

For KD analysis, 8-week-old male mice were fed for 3 days or 4 weeks and sacrificed in a fed state. KD consisted 76% fat, 8.8% protein, and 0.74% carbohydrates (no sucrose) (w/w)^24^. For adenoviral infection, 8-week-old male mice were infected with the indicated adenovirus at 5.0 × 10^8^ pfu/g body weight, following which samples were collected 6 days after infection from mice in a fed state. For the fasting and re-feeding experiments, mice were fasted for 24 h and then fed a high-sucrose/fat-free diet for 12 h, as previously described^42^. All animal husbandry procedures and animal experiments were performed in accordance with the Regulation of Animal Experiments of the University of Tsukuba and were approved by the Animal Experiment Committee, University of Tsukuba.

### Histological analysis

Mouse livers were fixed, embedded in paraffin, sectioned, and stained with haematoxylin and eosin, and Masson trichrome.

### Immunohistochemistry

Mouse livers were fixed in 10% formalin and embedded in paraffin blocks. The liver sections were then deparaffinized, and the antigens were retrieved by heating at 80 °C for 20 min. Following blocking, the sections were incubated with the primary antibodies for CD11b (1:600) (abcam, ab75476). After washing the sections with phosphate buffered saline (PBS), Histostar™ mouse (Medical & Biological Laboratories Co. Ltd. Japan) was applied at room temperature for 30 min, following which the samples were again washed with PBS. The reaction product was visualized by applying diaminobezidine (Dako Japan) for 5 min and then counterstaining the sections with haematoxylin.

### Cell culture

Mouse AML12.2 hepatoma cells were maintained in Dulbecco’s Modified Eagle Medium/Ham’s F12 media supplemented with ITS Liquid Media Supplement (SIGMA) and 10% fetal calf serum. Cells were infected with adenoviral green fluorescent protein (GFP) or the active form of CREB3L3 at a multiplicity of infection of 100 and then incubated for 24 h.

### Ketogenesis assay

To measure ketogenic activities *in vivo*, 8-week-old male mice were administered 0.5 M sodium octanoate at a dose of 6 μl per gram of body weight after 18 h of fasting^27^. Blood samples were collected 0, 1, 2, and 4 h after the injection, and plasma β-OH butyrate levels were determined using the β-OH butyrate colorimetric assay (Sanwa kagaku).

### Plasmids

The active form of human *CREB3L3* (1–320 aa) (AB0050902) was cloned using PCR into the pcDNA3 vector. The mouse *Cpt1a* promoter from −2200 bp to −1480 bp was cloned using PCR and subcloned into the pGL3-promoter luciferase vector (Promega). The mouse *Bdh1* promoter from −720 bp to +130 bp and the mouse *Ppara* promoter from −480 bp to +100 bp were cloned using PCR and subcloned into the pGL3-basic luciferase vector (Promega).

### Preparation of recombinant adenovirus

The cDNAs coding the active form (1–320 aa) of human *CREB3L3* and GFP were cloned into pShuttle vectors (Clontech). In addition, the recombinant adenovirus vectors were recombined with pAdeno-X vectors (Clontech). The recombinant adenoviruses were produced in 293A cells (Invitrogen) and purified using CsCl gradient centrifugation, as previously described^43^.

### Metabolic measurements

The levels of glucose, insulin, TG, FFA, TC, AST, ALT, and β-OH butyrate in the plasma, and the levels of glycogen, TG, and TC in the liver were measured as previously described^43^. Plasma FGF21 levels were measured using mouse FGF21 Quantikine® ELISA kits (R&D systems).

### Immunoblotting

Total cell lysates were immunoblotted as previously described^43^, using antibodies to α-tubulin (Millipore) and an anti-mouse CREB3L3 polyclonal antibody, which was generated as previously described^24^.

### Analysis of gene expression

Total RNA from cells and tissues was prepared using TRIzol® Reagent (Invitrogen). Real-time PCR analysis was performed using total RNA for cDNA synthesis (Invitrogen) with the ABI Prism 7300 system (ABI) and SYBR® Green Master Mix (Roche)^44^. Primer sequences are available upon request.

### Promoter analysis

Mouse AML12.2 hepatoma cells were transfected with the indicated luciferase reporter, expression plasmids, and a pRL-SV40 plasmid as a reference (Promega) using FuGENE6 (Roche). After a 24-h incubation, the firefly luciferase activity was measured and normalized to the *Renilla* luciferase activity. We generated CREB3L3 from an expression vector using an *in vitro* reticulocyte transcription–translation system (Promega). We used the following sequences in the EMSAs: 5′-gaaaacctggtgacgttggctgagcaaata-3′ for the WT of the *Cpt1a* promoter; 5′-gaaaacctggt**a**ac**c**ttggctgagcaaata-3′ for the Mut of the *Cpt1a* promoter; 5′-gcttactttcctggccttgctcagggttctctgg-3′ for approximately −200 to −166 of the *Bdh1* promoter; 5′-gttctctggctgtgtgtgtgtgtgtgtgtgtcc-3′ for −175 to −141 of the *Bdh1* promoter; 5′-gtgtgtgtcccttagctgcagcgtctacccttgat-3′ for −151 to −117 of the *Bdh1* promoter; and 5′-gtctacccttgatcttctgcttgccagagggt-3′ for −126 to −98 of the *Bdh1* promoter. We incubated the *in vitro*-translated protein lysate in a reaction mixture as previously described^43^ and resolved the DNA-protein complexes on a 4% polyacrylamide gel.

### Chromatin immunoprecipitation (ChIP) assay

ChIP assays were performed as previously described with some modifications^43^. In brief, we collected the livers of normal mice in fasted for a 24 h and fed states. AML12.2 cells were infected with GFP or the active form of CREB3L3 tagged with HA in the N-terminus at a multiplicity of infection of 100 and then incubated for 48 h. Minced liver tissues and AML12.2 cells were fixed in 1% formaldehyde for 15 min at room temperature. The soluble chromatin was subjected to immunoprecipitation with anti-mouse CREB3L3 polyclonal antibody^24^, anti-HA (Y-11, Santa Cruz), or with control IgG, and rotated overnight at 4 **°**C. Immune complexes were washed and then incubated overnight at 65 **°**C for reverse crosslinking. Chromatin DNA was extracted with phenol-chloroform, precipitated with ethanol, resuspended in water, and subjected to real-time PCR analysis. The primers used for real-time PCR were as follows: *Cpt1a* promoter region containing the CREB3L3 binding site, 5′-cgttggcagccttgggtttg-3′ and 5′-acacgttttgagtcaatatcggaggtag-3′; *Cpt1a* distal region, 5′-tgctctgtgaaagatgcttatg-3′ and 5′-cacactggccccaagcca-3′; *Bdh1* promoter region containing the CREB3L3 binding site for mouse liver, 5′-gcttactttcctggccttgctcagggtt-3′ and 5′-accctctggcaagcagaagatcaagggt-3′; *Bdh1* promoter region containing the CREB3L3 binding site for AML12.2 cells, 5′-gttcccagcatgccagaca-3′ and 5′-gaatttgaccctctggcaag-3′; *Bdh1* negative region, 5′-ccgtgtaacctcgaaactgc-3′ and 5′-gtgcatgctgagcgagcac-3′.

### Statistical analyses

Statistical significance was calculated using unpaired Student’s *t*-tests, one-way ANOVA followed by Tukey-Kramer test, or repeated-measures two-way ANOVA with Bonferroni post hoc t-test with a significance level of *P* < 0.05. All data are expressed as mean ± SEM.

## Additional Information

**How to cite this article**: Nakagawa, Y. *et al*. CREB3L3 controls fatty acid oxidation and ketogenesis in synergy with PPARα. *Sci. Rep.* **6**, 39182; doi: 10.1038/srep39182 (2016).

**Publisher's note:** Springer Nature remains neutral with regard to jurisdictional claims in published maps and institutional affiliations.

## Supplementary Material

Supplementary Figure
