# Supplementary material for: CREB3L3 controls fatty acid oxidation and ketogenesis in synergy with PPARα
Source: Sci Rep. 2016 Dec 16;6:39182. doi: 10.1038/srep39182 (PMC5159891; doi:10.1038/srep39182)
Supplement: Supplementary Figure [file srep39182-s1.pdf]

Supplementary Information

CREB3L3 controls fatty acid oxidation and ketogenesis in synergy with PPARα

[1,2,*]Yoshimi Nakagawa, [1]Aoi Satoh , [1]Hitomi Tezuka, [1]Song-iee Han, [1]Kenta Takei, [1]Hitoshi Iwasaki, [1]Shigeru Yatoh, [1]Naoya Yahagi, [1]Hiroaki Suzuki, [3]Yasumasa Iwasaki, [4]Hirohito Sone, [1]Takashi Matsuzaka, [1]Nobuhiro Yamada, [1,2,5,*]Hitoshi Shimano

[1]Department of Internal Medicine (Endocrinology and Metabolism), Faculty of Medicine, University of Tsukuba, Tsukuba, Ibaraki 305-8575, Japan
[2]International Institute for Integrative Sleep Medicine (WPI-IIIS), University of Tsukuba, Tsukuba, Ibaraki 305-8575, Japan
[3]Health Care Center, Kochi University, Kochi Medical School, Kochi, Kochi 780-8520, Japan
[4]Department of Hematology, Endocrinology and Metabolism, Niigata University Faculty of Medicine, Niigata, Niigata 951-8510, Japan
[5]Life Science Center, Tsukuba Advanced Research Alliance (TARA), University of Tsukuba, Tsukuba 305-8577, Japan

*Correspondence: ynakagawa@md.tsukuba.ac.jp (Y.N), hshimano@md.tsukuba.ac.jp (H.S.)

Fig. 1b

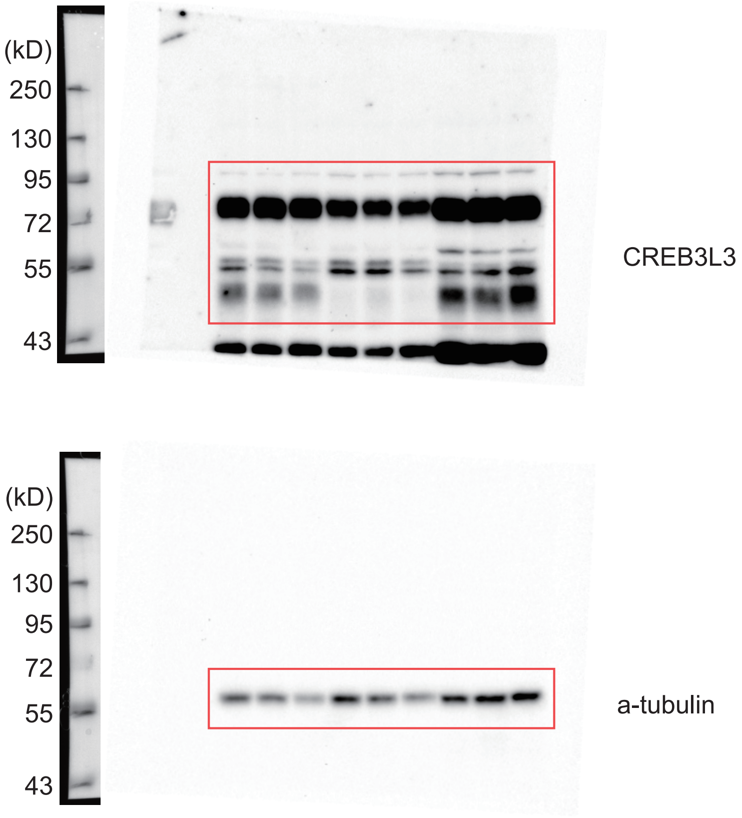

Supplementary Figure 1. Representative not cropped Western blots corresponding to Figure 1b.
